# Current knowledge of bone-derived factor osteocalcin: its role in the management and treatment of diabetes mellitus, osteoporosis, osteopetrosis and inflammatory joint diseases

**DOI:** 10.1007/s00109-024-02418-8

**Published:** 2024-02-16

**Authors:** Monika Martiniakova, Roman Biro, Veronika Kovacova, Martina Babikova, Nina Zemanova, Vladimira Mondockova, Radoslav Omelka

**Affiliations:** 1 Department of Zoology and Anthropology, Faculty of Natural Sciences and Informatics, Constantine the Philosopher University in Nitra, Tr. A. Hlinku 1, 949 01 Nitra, Slovakia; 2 Department of Botany and Genetics, Faculty of Natural Sciences and Informatics, Constantine the Philosopher University in Nitra, Tr. A. Hlinku 1, 949 01 Nitra, Slovakia

**Keywords:** Osteocalcin, Preclinical and clinical studies, Diabetes mellitus, Osteoporosis, Osteopetrosis, Inflammatory joint diseases

## Abstract

Osteocalcin (OC) is the most abundant non-collagenous and osteoblast-secreted protein in bone. It consists of two forms such as carboxylated OC (cOC) and undercarboxylated OC (ucOC). While cOC promotes bone mineralization and increases bone strength, ucOC is regarded an endocrinologically active form that may have several functions in multiple end organs and tissues. Total OC (tOC) includes both of these forms (cOC and ucOC) and is considered a marker of bone turnover in clinical settings. Most of the data on OC is limited to preclinical studies and therefore may not accurately reflect the situation in clinical conditions. For the stated reason, the aim of this review was not only to summarize current knowledge of all forms of OC and characterize its role in diabetes mellitus, osteoporosis, osteopetrosis, inflammatory joint diseases, but also to provide new interpretations of its involvement in the management and treatment of aforementioned diseases. In this context, special emphasis was placed on available clinical trials. Significantly lower levels of tOC and ucOC could be associated with the risk of type 2 diabetes mellitus. On the contrary, tOC level does not seem to be a good indicator of high bone turnover status in postmenopausal osteoporosis, osteoarthritis and rheumatoid arthritis. The associations between several pharmacological drugs used to treat all disorders mentioned above and OC levels have also been provided. From this perspective, OC may serve as a medium through which certain medications can influence glucose metabolism, body weight, adiponectin secretion, and synovial inflammation.

## Introduction

Osteocalcin (OC) is a major non-collagenous protein in bone synthesized by osteoblasts (bone-forming cells), but also by odontoblasts and hypertrophic chondrocytes [1]. It contains 49 amino acids in humans; while in mice it is made up of 46 amino acids. Overall, OC consists of two forms, such as carboxylated (cOC) and undercarboxylated (ucOC), and can be measured in the serum separately or as a total OC (tOC) [2, 3], which includes both aforementioned forms as well as detectable fragments released during bone resorption [4].

**Fig. 1:**
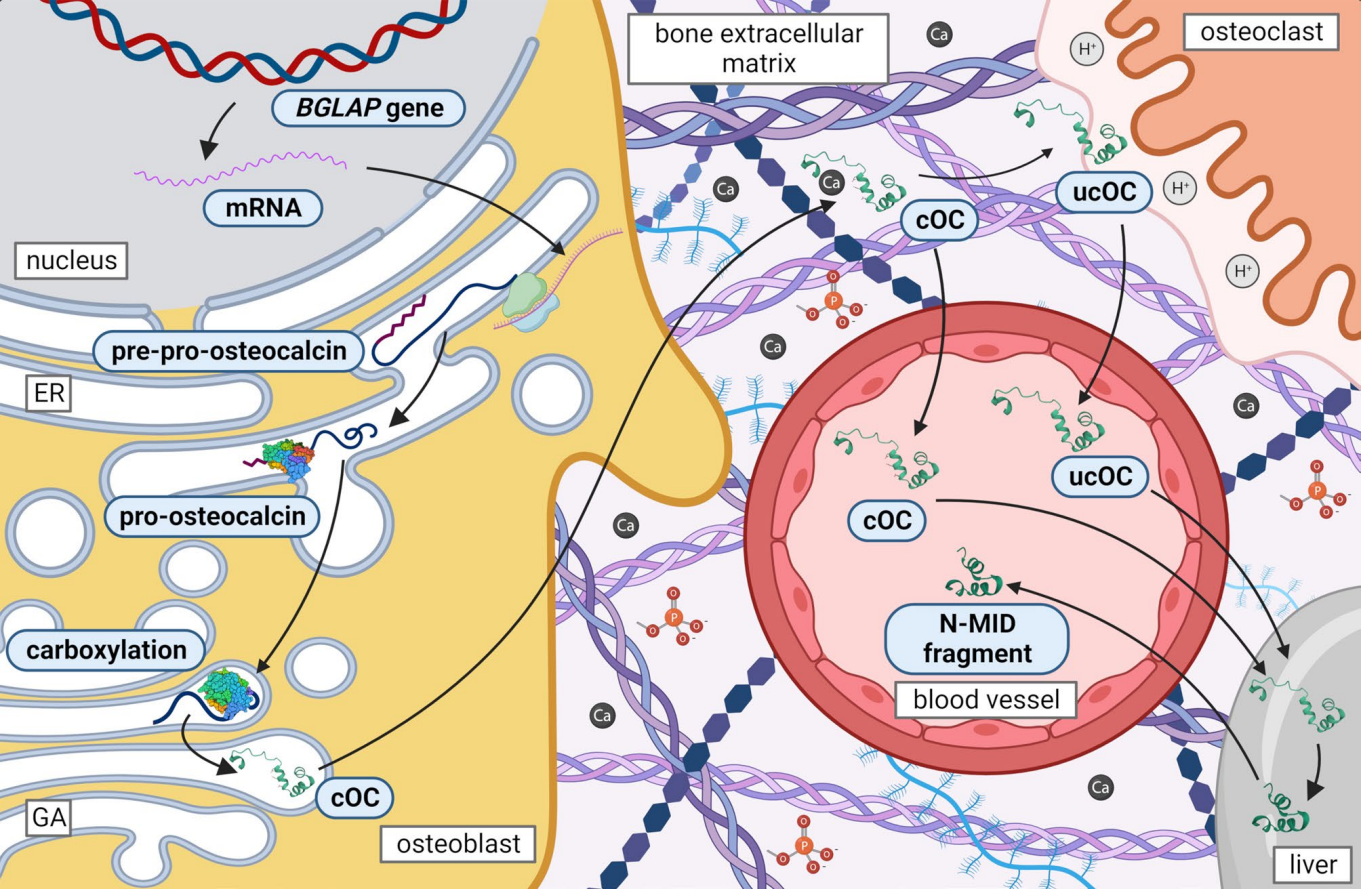
OC biosynthesis and metabolism. Expression of the *BGLAP* gene produces pre-pro-osteocalcin, which contains a signal sequence, a pro-peptide, and a chain. The signal sequence is removed in the endoplasmic reticulum to form pro-osteocalcin containing a propeptide and an osteocalcin chain. Three glutamate residues located in the Gla domain can be carboxylated by γ-glutamyl carboxylase. After cleavage of the pro-peptide by proprotein convertase, mature OC is finally formed and secreted into bone microenvironment. During bone resorption by osteoclasts, the acidic pH causes decarboxylation and ucOC can be released into the systemic circulation. Circulating OC is degraded in the kidney and liver to a more stable N-MID-fragment, so intact and fragmented molecules coexist in the serum (created with BioRender.com)

When bone is resorbed by osteoclasts (bone-resorbing cells), the acidic pH in the resorption lacuna causes the carboxyl groups on OC to be removed and ucOC to be released into the systemic circulation (Fig. 1). Therefore, circulating levels of ucOC are dependent on the rate of bone turnover (remodeling) [2, 5]. In general, serum levels of tOC, cOC, and ucOC can elevate with increasing bone formation, while ucOC level also elevates with increasing bone resorption [6].

The ucOC/tOC ratio is often measured in clinical studies. This parameter is higher in older adults compared to young individuals [7]. Interestingly, increased ucOC/tOC ratio was associated with reduced muscle function and elevated risk of long-term fall-related hospitalizations in elderly women, who had worse physical function, mobility, and a greater fear of falling. According to Luukinen et al. [8], higher ucOC/tOC ratio may also predict fracture risk in older men.

Overall, ucOC represents approximately one third of tOC. The serum tOC level (less than 50 ng/ml in mice and <30 ng/ml in humans) is considered a bone turnover marker in clinical conditions and reflects the osteoformation ability of osteoblasts. In mice, OC levels peak during the day and are lowest at night, whereas in humans, levels are very low in the morning, begin to increase in the afternoon, and peak at night [2, 9, 10]. These alterations may be consistent with modulation of glucocorticoid levels. Therefore, when extrapolating results from mouse studies to the human settings, the time point at which samples are collected should also be taken into account [11].

**Fig. 2:**
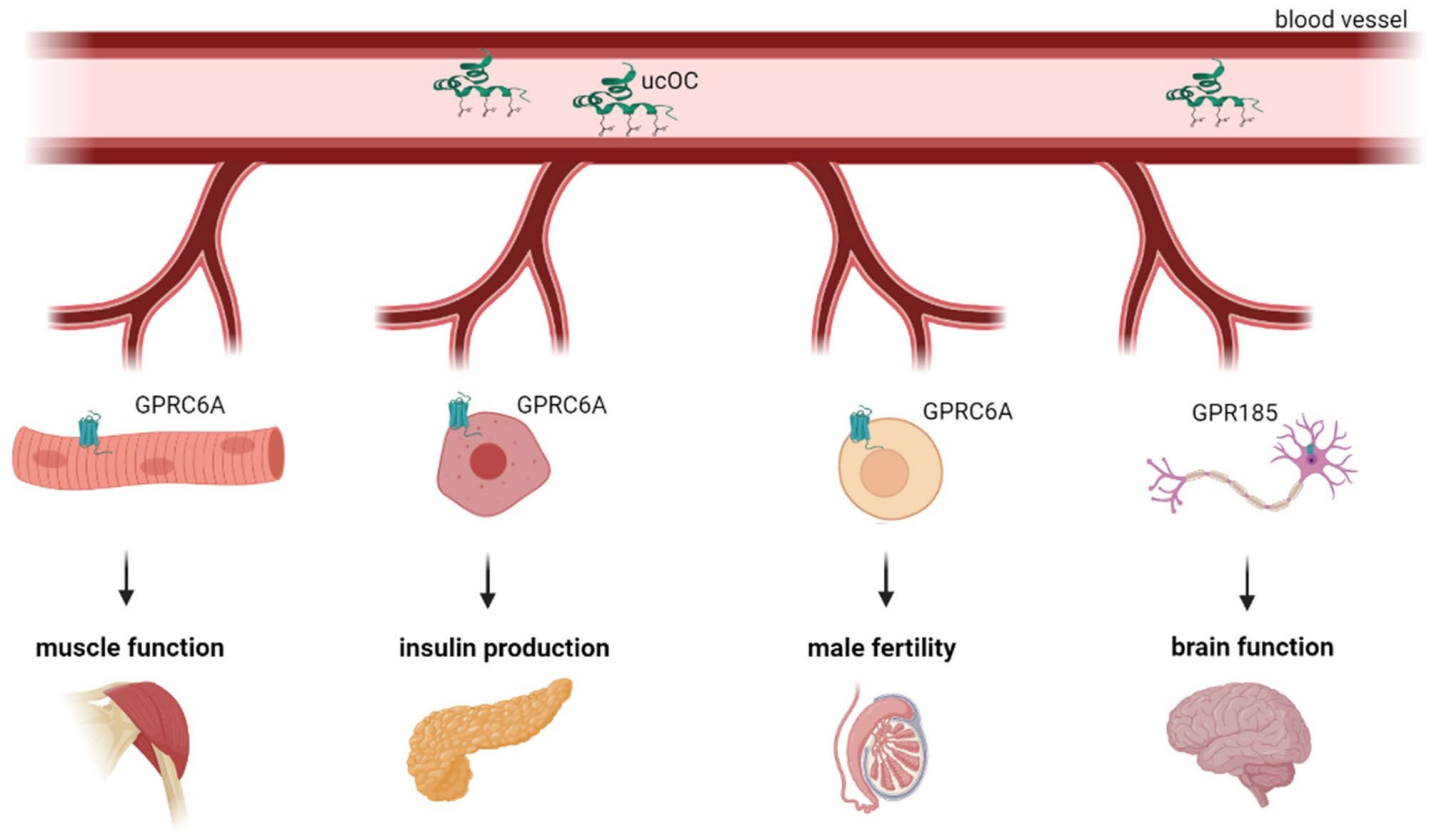
Schematic representation of the putative functions of ucOC. ucOC is released into the systemic circulation, binds to receptors on the cell surface (GPRC6A receptors are found in muscles, pancreas, testicles and GPR158 receptor is present in the brain) and can regulate muscle function, insulin production, male fertility and brain function (created with BioRender.com)

Generally, ucOC is regarded an endocrinologically active form of OC in preclinical studies [12]. The specific receptor of ucOC is G protein-coupled receptor class c group 6 member A (GPRC6A) [13]. It is broadly expressed in various organs, with the exception of the brain [14–17]. The G protein-coupled receptor 158 (GPR158) acts as ucOC receptor in the brain, and is associated with cognitive function ([18]; Fig. 2).

Based on various investigations, ucOC has been found to possess multiple functions, including increasing insulin secretion and proliferation of pancreatic β cells as well as adiponectin secretion from adipose tissue, thereby improving insulin sensitivity and reducing fat mass [19], improving skeletal muscle exercise capacity [16], enhancing testosterone secretion from testis [14], increasing glucagon-like peptide-1 (GLP-1) secretion from the intestine [15], enhancing cognitive function in the brain [20]. These various effects of ucOC have been suggested to be closely related to glucose metabolism and diabetic complications [10]. Therefore, ucOC has been proposed to act as a hormone (osteokine) with pleiotropic effects in multiple end organs and tissues, e.g. muscles, pancreas, male gonads, brain (Fig. 2) to regulate muscle mass, glucose and energy metabolism, male fertility, cognitive functions and behavior [10, 21]. The link between the skeleton and exoskeletal organs mentioned above demonstrates the importance of the endocrine function of the skeletal system. However, it is important to keep in mind that most of the data obtained has been limited to *in vitro* and animal model studies.

In general, OC can be clinically detected using enzyme immunoassays, chemiluminescent immunoassays or radioimmunoassays. Circulating OC has a short half-life (about 5 minutes) and is degraded in the kidney and liver, so intact and fragmented segments coexist in the serum (Fig. 1). The heterogeneity of these fragments is believed to limit its use [22]. Thus, tOC consists especially of intact OC and N-MID (N-terminal mid)-fragment. While intact OC is unstable due to protease cleavage between amino acids 43 and 44, the N-MID-fragment resulting from the cleavage, is considerably more stable. Therefore, assays usually detect a stable N-MID-fragment [23, 24]. However, assays detecting both the intact OC polypeptide and the N-MID-fragment may also be used [25–27]. The ucOC can be measured using hydroxyapatite binding assay (uses hydroxyapatite to bind cOC that is removed by centrifugation, and ucOC is subsequently measured) and direct immunoassays (use antibodies against ucOC; however, commercially available ucOC antibody can overestimate large ucOC fragments, leading to inaccuracies in the determination of ucOC or ucOC/tOC ratio) [4, 28, 29]. For this aim, measurement of ucOC is not straightforward, and frequently used methods do not distinguish the number and position of non-carboxylated glutamate residues, and these limitations should be considered when interpreting the results [4]. In any case, methods for the determination of circulating ucOC are nowadays gradually increasing [30], but they need to be optimized for routine use.

Despite the initial search for information on OC and the subsequent abundance of articles, to our knowledge, current information on OC with respect to all its forms (cOC, ucOC, tOC) as well as its role in most common bone-related disorders is scattered. Recent controversies regarding the proposed role of OC in skeletal development, energy metabolism, male fertility, and nervous system have been characterized by Komori [31], Manolagas [5], Wang et al. [32] primarily using animal models. Information obtained from clinical studies has not been provided. Several authors evaluated the associations of serum OC level with diabetes mellitus [2, 21, 33]; however, knowledge of which type of OC (tOC or ucOC) was analysed in patients, was not often specified. Relationships between OC levels and other bone-related diseases appear sporadically in a few articles [1, 11, 34], but they are not summarized in a dedicated review. Therefore, the main aim of this review was not only to summarize current knowledge about all forms of OC, but also to provide new interpretations of its involvement in the management of diabetes mellitus, osteoporosis, osteopetrosis and two inflammatory joint diseases (osteoarthritis and rheumatoid arthritis). In this context, special emphasis was placed on available clinical studies and accurate information on the type of OC under investigation. Less well-known associations between pharmacological treatment of all aforementioned disorders and OC levels were also provided to demonstrate the potential of OC to serve as a medium through which certain medications can influence some important indicators of a particular disease.

## Molecular structure of osteocalcin

**Fig. 3:**
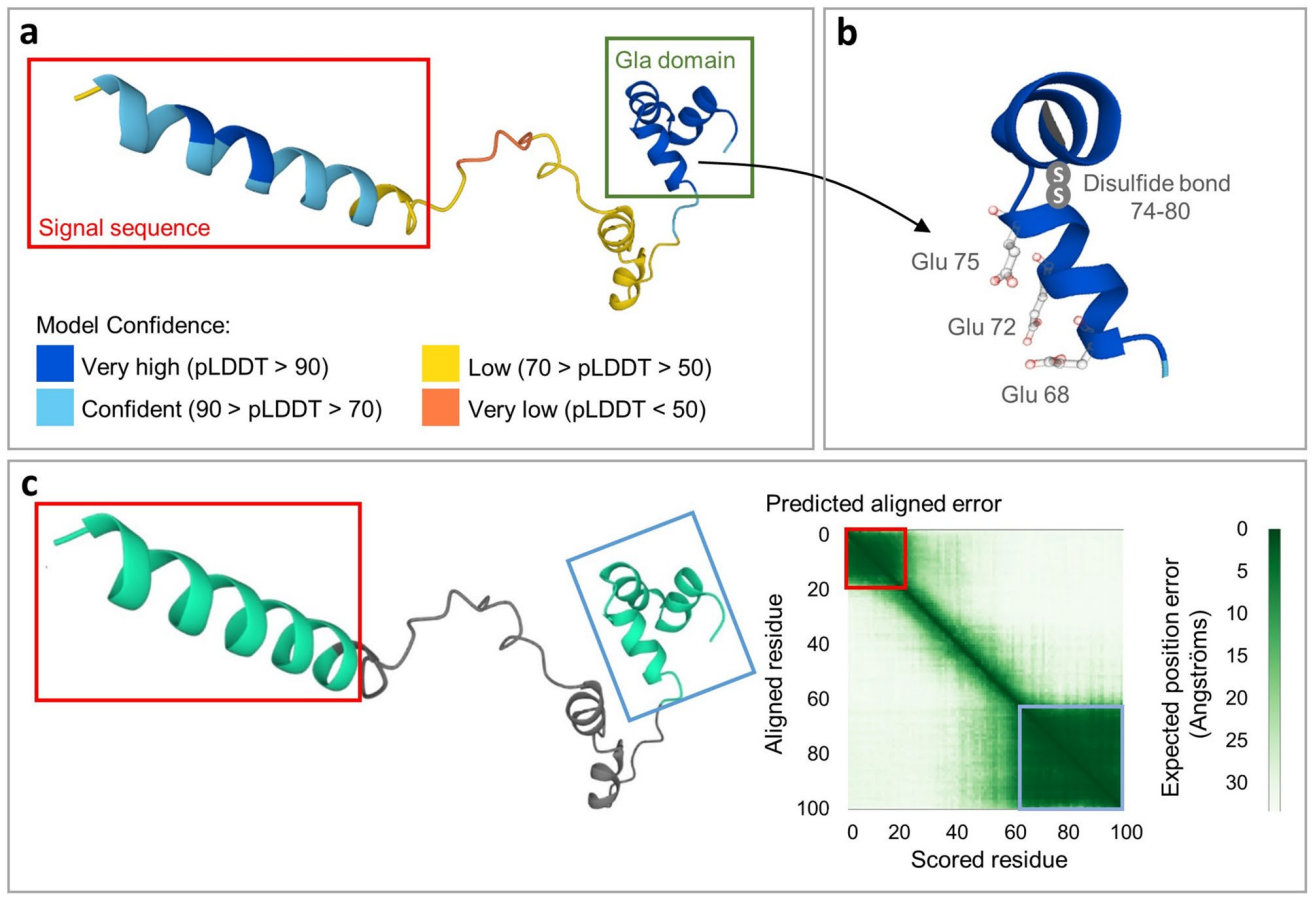
Human OC structure prediction according to AlphaFold Protein Structure Database [168, 169]. **a** – 3D visualization of OC structure prediction with colored per-residue confidence metric (pLDDT). The structures of the signal sequence (red box) and Gla domain (green box) show the highest confidence score. **b** - Positions of carboxylation and disulfide bond on the OC molecule (Gla domain). **c** - Inter-domain (inter-structure) arrangement prediction with predicted aligned error. The two dark green (low-error) squares on the 2D plot correspond to the two distinct domains (structures) highlighted in red and blue boxes. The alignment of the middle sequence (shown in gray) relative to the other residues shows a high error

**Fig. 4:**
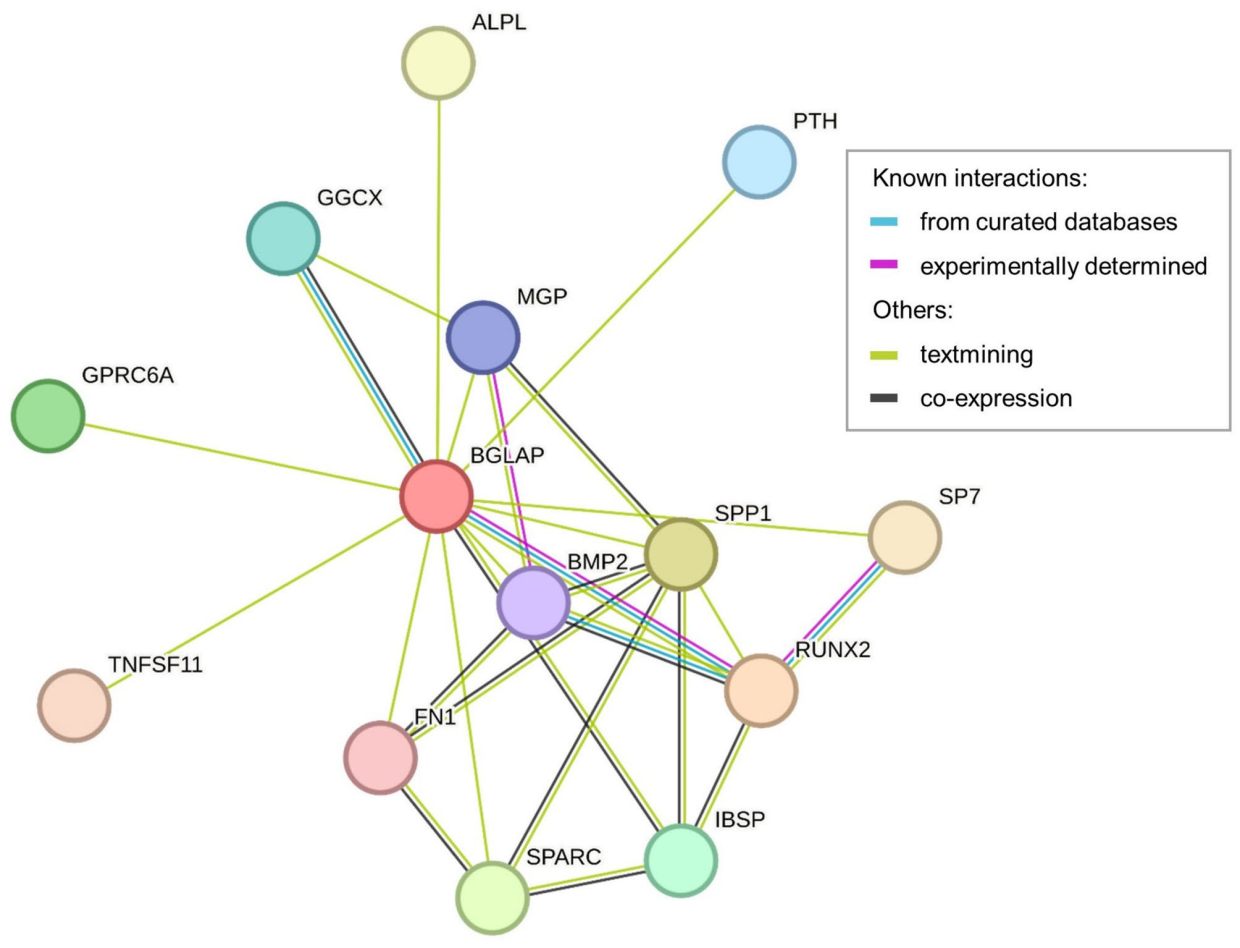
Predicted interactions and functional partners of OC. The network comes from STRING, a database of known and predicted protein interactions [170]. The evidence view with a minimum interaction score of 0.9 (the highest confidence) is displayed. Different line colors represent the types of evidence for the association. ALPL - Alkaline phosphatase, tissue-nonspecific isozyme; BGLAP – Osteocalcin; BMP2 - Bone morphogenetic protein 2; FN1 – Fibronectin; GGCX - Vitamin K-dependent gamma-carboxylase; GPRC6A - G-protein coupled receptor family C group 6 member A; IBSP - Bone sialoprotein 2; MGP - Matrix Gla protein; PTH - Parathyroid hormone; RUNX2 - Runt-related transcription factor 2; SP7 - Transcription factor Sp7; SPARC – SPARC; SPP1 – Osteopontin; TNFSF11- Tumor necrosis factor ligand superfamily member 11, membrane form

In humans, OC is encoded by a single *BGLAP* (bone gamma-carboxyglutamate protein) gene, located on chromosome 1 at 1q25-q31. The pre-pro-osteocalcin molecule with a length of 100 amino acids is first synthesized by gene expression (UniProtKB AC P02818; [35]). The predicted structure of the pre-pro-osteocalcin molecule with the confidence of prediction of individual parts is shown in Fig. 3a, c. Like most secreted proteins, OC has a signal sequence (amino acids 1-23) that is removed in the endoplasmic reticulum to form pro-osteocalcin containing a 28 amino acid pro-peptide and a 49-residue osteocalcin chain. The molecule contains Gla domain with three glutamate residues at positions 68, 72 and 75 that can be carboxylated (Fig. 3b), which then provide high affinity to the hydroxyapatite matrix. This modification is catalyzed by γ-glutamyl carboxylase, and uses vitamin K, O_2_, and CO_2_ as cofactors, supplied by the vitamin K cycle and circulation [11, 36]. In bone, cOC represents 15% of non-collagenous matrix proteins [2] and is essential for the alignment of apatite crystals and optimal bone strength. Bone strength is elevated by cOC [31]. The ucOC with a reduced degree of carboxylation at the glutamate residues (carboxylation is missing at one or more positions) is available with less affinity for hydroxyapatite [11]. Mature OC consisting of 49 residues is finally formed by cleavage of the pro-peptide by the proprotein convertase furin and secreted into the bone microenvironment ([37]; Fig. 1). The OC is able to interact with many molecules that have an important function in bone physiology. Predicted interactions and functional partners of OC are illustrated in Fig. 4.

## Osteocalcin-deficient animal models

Due to considerable differences between mouse and human OC at both the genomic and protein level, the validity of extrapolating findings from the OC-deficient mouse model to human disease has been questioned [38]. In general, mice have a gene cluster of OC consisting of Bglap, Bglap2, and Bglap3 genes within a 23 kb span of genomic DNA, while one OC gene has been determined in rats and humans [39]. Bglap and Bglap2 are specifically expressed in bone, while Bglap3 is expressed in non-osteoid tissues (e.g. kidney, male gonadal tissues, lung) [40, 41]. OC expression is regulated by runt-related transcription factor 2 (Runx2), which is an essential transcription factor for osteoblast differentiation [42]. In fact, Runx2 deficient mice do not express any OC; its expression was reduced by Runx2 antisense oligonucleotides in rat primary osteoblasts and ROS17/2.8 osteoblastic cells, and overexpression of Runx2 induced OC expression in C3H10T1/2 multipotent mesenchymal cells [43–46].

Ducy et al. [47], generated the first OC-deficient mice (in 129Sv:C57BL/6J mixed genetic background) that had significantly elevated trabecular and cortical bone volume, cortical bone thickness, bone mineral density (BMD), and bone strength. The number of osteoclasts and bone marrow area were higher in OC-null mice. In addition, ovariectomy raised bone marrow area and reduced bone strength in this mouse model. In accordance with the findings of aforementioned study, OC has been considered a negative factor of bone formation that reduces osteoblast function and osteoclastogenesis. Moreover, OC-null mice displayed impaired glucose metabolism [28], reduced testosterone synthesis [14], and muscle mass [16]. However, the new OC-deficient mice generated by Diegel et al. [48], (in C57BL/6J,C3H mixed genetic background, the authors replaced DNA encoding Bglap and Bglap2 with a neo cassette in embryonic stem cells) and Moriishi et al. [6] (in C57BL/6N mixed genetic background, the authors used CRISPR/Cas9-mediated gene editing to remove most of the Bglap and Bglap2 protein coding regions) did not confirm the inhibition of bone formation by OC. Consistent with their findings, OC did not affect bone formation, bone resorption, and bone mass in either estrogen-deficient or estrogen-sufficient conditions. Moreover, OC was not physiologically involved in glucose metabolism, testosterone synthesis or maintenance of muscle mass. Possible explanations for these discrepancies may include modifier genes, genetic background of mice, differences in the molecular genetics of knockout alleles, sex, or breeding environment. However, both studies mentioned above [6, 48] have brought a general consensus that OC promotes bone mineralization. The rat OC gene locus shares greater synteny with the human locus. Therefore, Lambert et al. [38] generated OC-null mutant rats (using the CRISPR/Cas9 system) and reported increased trabecular thickness, BMD, and trabecular bone volume. In contrast to the findings of Ducy et al. [47], their data did not demonstrate elevated cortical bone volume and density. Also, OC-deficient rats did not develop obesity, insulin resistance, or glucose intolerance. Further investigations are therefore necessary to clearly determine the causal effect of OC.

## Osteocalcin and diabetes mellitus

Diabetes mellitus (DM) represents a worldwide public health issue affecting approximately 450 million adults [49]. In addition to the most frequent secondary complications of DM (including diabetic retinopathy, nephropathy, neuropathy, cardiovascular diseases) [50], diabetic bone disorder is often diagnosed, which justifies the inclusion of DM among bone-related diseases. It is manifested by altered bone mineral density (BMD), abnormalities in bone metabolism and microarchitecture, reduced bone strength. Although both type 1 diabetes mellitus (T1DM) and type 2 diabetes mellitus (T2DM) share common features, notably hyperglycemia, the etiology of diabetic bone disorder differs between them. While reduced BMD is mainly determined in T1DM, the BMD may not be affected in T2DM patients. However, a higher risk of fractures is demonstrated in both T1DM and T2DM [51, 52].

Strong evidence suggests that ucOC could exert its metabolic effects by targeting multiple tissues essential for glucose and lipid metabolism. In the pancreas, ucOC can promote β-cell proliferation and insulin production through GPRC6A [19, 53]. The ucOC can also elevate delta like-1 (DLK1) production in pancreatic β cells, and DLK1 inhibits the insulin signaling-dependent OC production in osteoblasts [54]. Therefore, there is a positive feedback loop between pancreatic islets and bone. The ucOC can also indirectly favor insulin production by increasing GLP-1 secretion from the intestine [15]. In insulin target tissues, ucOC can increase glucose and fatty acid uptake [16, 55], insulin sensitivity [56], nutrient utilization and mitochondrial capacity [16] and reduce glycogen production in muscle and lipid synthesis in liver [16, 57].

It has been reported that circulating levels of ucOC are decreased in both humans and mice in the presence of metabolic syndromes such as insulin resistance and DM [58–60] and that these disorders can be alleviated by ucOC administration [28, 60–62]. Obese diabetic mice administered ucOC have been shown to improve systemic glucose tolerance and insulin sensitivity, concomitantly with reductions in hyperlipidemia and whole-body adiposity [61–63]. Accordingly, the overexpression of ucOC protected mice from obesity and glucose intolerance [28]. In insulin resistant tissues, ucOC treatment restored the impaired response to insulin stimulation, disturbed energy metabolism and reduced mitochondrial capacity [19, 60, 62, 64].

In patients with metabolic syndrome, lower tOC levels were reported compared to controls, and increases in tOC levels were associated with decreases in fasting plasma glucose, glycated hemoglobin (HbA1c), insulin resistance index (HOMA-IR) and body mass index (BMI) [58]. According to Liang et al. [65], tOC level was the highest in the normal glucose tolerance group and gradually decreased in the impaired glucose tolerance group and T2DM participants. After a four-year follow-up, group with low tOC (<23.33 ng/mL) showed an increased risk of T2DM, reduced fasting glucose and insulin resistance (IR). Many studies also confirmed significantly reduced tOC levels in individuals with T2DM versus healthy controls [65–76]. On the other hand, a minimal number of studies did not reveal any differences [77, 78]. The data are summarized in Table 1. According to Zeng et al. [79], high tOC was associated with lower blood glucose, IR, triglycerides and BMI. Thus, OC may be positively correlated with glycemic metabolic status, and reduced level of serum tOC could be consistent with incident T2DM.

**Table 1:** Summary table of OC levels in patients with bone-related diseases and healthy controls

**Disease**	**Patients with the disease**	**Healthy controls**	**P-value**	**Ref.**
type 2 diabetes mellitus tOC (ng/ml)	2.5 ± 1.24	4.4 ± 1.32	< 0.01	[66]
	4.44 ± 3.53	8.82 ± 4.03	< 0.001	[67]
	15.3 ± 4.1	18.3 ± 5.3	0.012	[68]
	17.5 ± 6.4	22.2 ± 9.4	< 0.005	[69]
	15.1 (10.8–18.3)	16.8 (11.8–20.6)	< 0.05	[70]
	21.7 ± 8.2	27.8 ± 13.8	< 0.001	[71]
	10.2 ± 0.2	12.8 ± 0.1	< 0.05	[72]
	3.6 ± 1.5	5.7 ± 1.1	NS	[77]
	18.56 ± 19.47	21.21 ± 10.85	< 0.001	[73]
	18.58 (15.75–22.59)	23.6 (19.24–29.23)	< 0.001	[65]
	7.30 (5.00–9.82)	12.2 (7.12–17.4)	0.001	[75]
	18.1 (14.2–22.9)	21.8 (17.2–27.8)	< 0.001	[76]
type 2 diabetes mellitus ucOC (ng/ml)	3.57 (2.33–5.35)	4.45 (2.62–6.47)	< 0.05	[83]
	9.58 ± 6.29	11.22 ± 4.73	< 0.001	[73]
	3.04 ± 0.28^a^	4.48 ± 0.57	0.025	[84]
	1.5 ± 1.4	2.3 ± 1.8	< 0.05	[85]
	2.76 ± 0.38	4.52 ± 0.06	0.009	[30]
type 2 diabetes mellitus cOC (ng/ml)	7.53 (5.75–9.45)	8.48 (6.49–11.53)	< 0.001	[83]
osteoporosis tOC (ng/ml)	8.3 ± 6.0	10.5 ± 4.3	NS	[122]
	6.06 ± 0.97	3.51 ± 0.42	< 0.01	[113]
	28.0 ± 12.5^b^	27.5 ± 11.2	NS	[123]
	10.6 ± 5	7.3 ± 3.6	< 0.05	[114]
	5.03 ± 0.56	2.61 ± 0.55	< 0.001	[115]
	9.85 ± 2.72^c^	9.67 ± 3.37	NS	[124]
	13.0 ± 5.3	9.2 ± 3.5	< 0.05	[116]
	15.3 ± 8	7.9 ± 3.6	< 0.001	[117]
	30.24 ± 12.32	23.95 ± 9.27	< 0.01	[118]
	26.13 ± 15.35	24.08 ± 16.08	< 0.001	[119]
	6.0 (3.5–8.5)	12.4 (9.4–17.8)	< 0.001	[125]
	43.5 (36.5–64.0)	22.5 (18.2–28.5)	0.001	[121]
osteoporosis ucOC (ng/ml)	6.7 ± 4.8^b^	5.8 ± 4.1	NS	[123]
	1.82 ± 1.76^c^	3.09 ± 3.94	< 0.05	[124]
	2.19 (2.11–4.02)	1.31 (0.86–1.75)	0.01	[121]
osteopetrosis ucOC/tOC	10.5 ± 4.80	39 ± 17.16	< 0.05	[150]
osteopetrosis tOC in bone (mg/kg)	51.4 ± 3.9	38.0 ± 3.6	< 0.03	[149]
destructive osteoarthritis tOC (ng/ml)	10.27 ± 3.47	8.20 ± 3.9	NS	[158]
non-destructive osteoarthritis tOC (ng/ml)	3.83 ± 1.59	7.42 ± 3.04	< 0.001	[158]
osteoartritis ucOC (ng/ml)	5.66 ± 4.70	< 4.5	< 0.05	[159]
rheumatoid arthritis tOC (ng/ml)	5.56 ± 3.67	6.09 ± 2.54	NS	[158]
	1.6 ± 0.4	3.3 ± 0.3	< 0.01	[164]

The values are expressed as mean ± standard deviation or median (interquartile range)

^a^prediabetic patients

^b^osteoporotic patients with hip fracture

^c^osteoporotic patients with diabetes

Large epidemiological studies indicated that a higher level of ucOC was associated with a lower risk of DM in the community [73, 80]. Bullo et al. [81] revealed that an increase in ucOC was consistent with a decrease in HOMA-IR over two years in a community-dwelling population. However, a prospective investigation showed no evidence of an association between ucOC and incident T2DM in elderly participants [82]. On the contrary, further studies reported lower levels of ucOC in subjects with T2DM compared to controls [30, 73, 83–85]. The role of ucOC in T1DM remains uncertain [4].

Considering pharmacological treatment of DM, a recent meta-analysis by Hu et al. [86] revealed no significant impact of metformin (an insulin sensitizer) administration on tOC level. Similarly, vildagliptin (an incretin-based therapy) had no effect on tOC level even after 12 months of application [87]. Identical findings were also recorded in diabetic women with osteoporosis receiving metformin and sitagliptin (an incretin-based therapy) [88]. Application of thiazolidinediones (insulin sensitizers) such as pioglitazone and rosiglitazone in diabetic patients produced various effects on tOC levels, ranging from a decrease [89], through non-significant changes [90, 91] to an increase in tOC [92]. In several cases, it is possible to observe variability in the concentration of tOC over time according to the length of treatment with a given thiazolidinedione. When using rosiglitazone, no significant changes in tOC levels were found after 12 weeks; however, rapid decrease in tOC was sustained during the 2-year follow-up. In contrast, in the pioglitazone study, serum tOC first decreased at 6 months and then almost returned to baseline at 12 months. Both thiazolidinediones are peroxisome proliferator-activated receptor (PPAR) agonists. However, rosiglitazone is a specific PPARγ activator, while pioglitazone is not only a selective human PPARγ1 but also a weak human PPARα activator [93], which may result in differences in their effects. In addition, it is necessary to consider the limitations of individual studies, such as small sample sizes, different dosages or different proportions of men and women. The results of Namvaran et al. [92] deviate from the trend of decreasing tOC levels due to thiazolidinediones, the authors explain this fact by breaking IR, where the cause of increased tOC may be reduced IR in peripheral tissues and improved glucose tolerance. Regarding SGLT2 inhibitors (e.g. canagliflozin, empagliflozin), longer treatment time appears to be required to affect tOC levels (Table 2). In addition, OC may serve as a medium through which certain pharmacological drugs can affect glucose metabolism. It is known that bone resorption is closely related to glucose homeostasis. Specifically, high fasting plasma glucose levels were measured in osteoporotic women treated with medications inhibiting bone resorption, and there was a positive correlation between ucOC and urinary cross-linked N-telopeptides of type I collagen (NTx, bone resorption marker) [94, 95]. Accordingly, the use of antiresorptive drugs should increase the risk of IR and DM due to reduced levels of ucOC. However, epidemiological and clinical trials have shown that the administration of antiresorptive drugs was not consistent with changes in plasma glucose, IR and the development of DM, but was associated with a reduced risk of DM, especially with long-term treatment [96–101]. Urano et al. [74] conducted a study involving postmenopausal women; some of them were treated with bisphosphonates (antiresorptive drugs). According to their findings, tOC levels were significantly correlated with HbA1c levels. Furthermore, a decrease (<6.1 ng/mL) of tOC was associated with the future development of T2DM. However, there was no trend towards an increased incidence of T2DM in patients treated with bisphosphonates [74]. The findings by Mazzioti et al. [102] also suggested that bisphosphonate treatment does not affect glucose metabolism, although lower tOC levels were noted in bisphosphonate-treated patients. Lewis et al. [103] found that HbA1c did not change significantly, although tOC and ucOC levels were lower in elderly women after 1 year of calcium carbonate supplementation compared to untreated individuals. Serum levels of OC were measured by radioimmunoassay or immunoradiometric assay.

**Table 2:** Summary table of associations between selected drugs and OC levels in treated and untreated individuals

**Disease**	**Selected drugs**	**DT**	**OC level in treated** **individuals**	**OC level in untreated** **individuals**	**P-value**	**Ref.**
type 2 diabetes mellitus tOC (ng/ml)	metformin	12 m	7.0 ± 3.2	7.1 ± 3.1	NS	[91] ^e^
	pioglitazone	12 m	7.7 ± 3.8	8.0 ± 3.5	NS	[91] ^e^
		6 m	6.8 ± 3.0	8.0 ± 3.5	< 0.05	[91] ^e^
		12w	16.04 ± 13.84	7.29 ± 6.54	< 0.001	[92] ^e^
	rosiglitazone	12w	2.8 ± 0.4	3.5 ± 0.5	NS	[90] ^f^
		24 m	2.5 ± 1.9	3.4 ± 2.5	< 0.05	[89] ^f^
	vildagliptin	12 m	12.5 ± 5.2	12.5 ± 4.2	NS	[87] ^f^
	canagliflozin	52w	+ 10.1%^a^	-	< 0.05	[170] ^h^
	empagliflozin	3 m	1.63 ± 0.03	1.63 ± 0.02^b^	NS	[171] ^g^
	glucocorticoids	3 m	7.57 ± 1.0^c^	11.56 ± 1.0^c^	< 0.001	[172] ^e^
type 2 diabetes mellitus + osteoporosis tOC (ng/ml)	metformin	12w	16.4 ± 6.9	17.7 ± 7.8	NS	[88] ^f^
	sitagliptin	12w	14.75 ± 2.7	16.8 ± 8.1	NS	[88] ^f^
osteoporosis tOC (ng/ml)	bisphosphonates	12 m	9 (4–20)	14 (4–32)	< 0.001	[102] ^f^
	risedronate	12 m	4.8 ± 1.2	7.5 ± 2.3	< 0.001	[139] ^e^
	teriparatide	12 m	30 (10–54)	15 (3–40)	< 0.001	[102] ^f^
	denosumab	24w	13.12 ± 2.42	24.21 ± 3.72	< 0.001	[140] ^g^
osteoporosis ucOC (ng/ml)	parathyroid hormone	12 m	10.65 (7.02–16.69)	4.4 (2.9–6.9)	< 0.01	[141] ^g^
	alendronate	12 m	3.27 (1.99–4.61)	4.6 (2.8–6.5)	< 0.01	[141] ^g^
	risedronate	12 m	0.8 ± 0.4	1.0 ± 0.4	NS	[139] ^f^
osteoarthritis tOC (ng/ml)	salmon calcitonin	24 m	22.3 (15.8–31.4)	22.1 (15.8–31.0)^b^	NS	[162] ^h^
rheumatoid arthritis tOC (ng/ml)	low-dose corticosteroids	12 m	3.3 (0.7–6.0)	4.2 (1.4–5.8)^d^	NS	[166] ^e^
	non-steroidal anti-inflammatory drugs	12 m	4.7 (0.5–8.0)	4.0 (1.4 -6.5)^d^	NS	[166] ^e^
	tocilizumab	24w	47.42 ± 2.8	36.29 ± 2.14	< 0.001	[167] ^f^

The values are expressed as mean ± standard deviation or median (interquartile range)

*DT* duration of treatment

^a^increase relative to placebo

^b^placebo group, otherwise values before treatment (baseline) are in this column

^c^baseline comparison of patients according to the presence of diabetes mellitus

^d^matched controls

^e^serum levels of OC were measured by radioimmunoassay or immunoradiometric assay

^f^serum levels of OC/ucOC were measured by solid-phase two-site chemiluminescent immunometric assay, electrochemiluminescence assay or competitive chemiluminescence immunoassay

^g^serum levels of OC/ucOC were measured by enzyme-linked immunosorbent assay or solid phase enzyme amplified sensitivity immunoassay

^h^the OC measurement method was not specified in the publication

A low bone remodeling status has been observed in diabetic patients treated with glucocorticoids (GC) [104] and could be associated with higher BMD [105]. According to Florez et al. [75], reduced tOC levels during GC treatment represent a risk factor for the manifestation of DM. Thus, GC-treated patients with tOC levels <9.25 ng/mL presented a sixfold higher risk of DM (Table 2). However, of all bone turnover markers measured, only OC was significantly associated with increased chances of DM. Insignificantly lower values of bone alkaline phosphatase (ALP), serum procollagen type I amino-terminal propeptide (PINP), and cross-linked C-terminal and N-terminal telopeptides of type I collagen (serum CTx and urine NTx) were reported in subjects with DM. The study by van Bommel et al. [106] showed that GC decreased tOC and PINP levels in a dose-dependent manner and that these alterations were related to adverse effects of GC on glucose and lipid metabolism. Furthermore, the effect of teriparatide (anabolic drug) on serum HbA1c and fasting plasma glucose was investigated by Mazzioti et al. [102]. This therapy generally increases bone formation and thus OC values (Table 2). A significant decrease in serum HbA1c was determined in diabetic patients, suggesting that teriparatide could cause some improvement in glucose homeostasis [102, 107]. Future research may assess whether treatments with more profound effects on OC interfere with glucose metabolism.

## Osteocalcin and osteoporosis

Osteoporosis is the most common skeletal disorder affecting approximately 200 million individuals worldwide [108]. It is characterized primarily by reduced BMD, alterations in bone microstucture and elevated risk of fragility fractures. At the microstructural level, increased bone resorption and decreased bone formation occur simultaneously, leading to bone loss. Age is one of the main risk factors for primary type 1 (postmenopausal) osteoporosis. Primary osteoporosis of type 2 (senile) occurs after the age of 75 and is diagnosed in a ratio of 2:1 in women and men [109, 110].

Although serum tOC is widely used as a bone turnover marker to indicate high remodeling status in postmenopausal osteoporosis (PMO) [22, 111, 112], alterations in tOC level in individuals with PMO compared to healthy controls remain controversial. Several studies indicated a higher level of tOC in PMO cases [113–121]. However, there are also researches reporting the same or even lower tOC level in osteoporotic patients [122–125]. Biver et al. [126] and Liu et al. [127] performed meta-analyses to compare several markers of bone turnover and found no significant difference in tOC level between osteoporotic and healthy individuals. Therefore, tOC does not appear to be a good indicator of high bone turnover status in PMO unless new techniques for standardized assessment of circulating OC are used. Despite remaining issues with reference intervals and assay harmonization, tOC has been shown to be useful in elucidating the pharmacodynamics and efficacy of osteoporosis drugs in clinical trials [128].

Vergnaud et al. [123] revealed that a higher ucOC level predicted hip fracture risk independently of femoral neck BMD in older women. Conversely, tOC was not associated with hip fracture risk [124]. However, significant changes in ucOC level were observed in both osteoporotic and diabetic patients with osteoporosis (Table 1). According to Xu et al. [129], elevated ucOC level correlated with lower BMD at the lumbar spine, femoral neck, and total hip in both elderly women and men. Similar to these findings, Szulc et al. [130] and Emmaus et al. [131] reported that older women with abnormally high serum ucOC had lower BMD values at all sites. Cummings et al. [132] identified a 26% decrease of femoral neck BMD in patients with abnormal ucOC levels, which corresponded to a five- to sevenfold increase in hip fracture. Horiuchi et al. [124] found that ucOC levels were significantly higher in subjects with osteoporosis versus non-osteoporotic individuals (Table 1).

Interestingly, lower levels of dietary vitamin K are consistent with higher ucOC levels, while vitamin K supplements reduce ucOC levels [133]. Therefore, ucOC can be used as a marker to determine whether vitamin K preparations should be given to patients with osteoporosis [10]. Menatetrenone is the form of a synthetic vitamin K_2_ that is widely used in clinical practice. In the study by Shiraki et al. [134], serum level of ucOC was lower and cOC higher after 1 month of menatetrenone treatment. Differences in ucOC and cOC levels persisted during 6 months of the therapy. In addition, a higher tOC level was reported after 6 months of menatetrenone administration. Conversely, Jiang et al. [135] identified a reduction for both tOC and ucOC as well as the ucOC/tOC ratio after 12 months of menatetrenone treatment. A meta-analysis by Su et al. [136] demonstrated nonsignificantly reduced ucOC and a lower ucOC/tOC ratio in patients treated with menatetrenone (as part of combination therapy) compared to the placebo group. On the other hand, warfarin, an anticoagulant drug, inhibits vitamin K-dependent carboxylase, prevents post-translational carboxylation of factors in the coagulation cascade and OC, thereby increasing the levels of ucOC and lowering blood glucose levels in mice. However, warfarin also regulates OC gene expression, so warfarin treatment interferes with the interpretation of studies of this protein and its role in carbohydrate metabolism [137].

In this context, a clinical study by Fernandez-Real et al. [138] evaluated the effect of a high-calorie diet and regular physical activity on tOC levels. According to their findings, weight loss through diet and physical activity can cause increase in tOC level associated with changes in visceral fat mass.

Lower tOC levels were found in osteoporotic patients treated with bisphosphonates including risedronate, as well as in those treated with denosumab, a RANKL-binding monoclonal antibody [102, 139, 140]. On the other hand, treatment with teriparatide resulted in an increase in tOC [102]. The relationship between ucOC levels in postmenopausal osteoporotic women treated with parathyroid hormone (PTH) or alendronate (a bisphosphonate) and alterations in metabolic parameters were investigated by Schafer et al. [141]. The ucOC levels were increased after PTH and decreased after alendronate administration (Table 2). The ucOC/tOC ratio was increased with PTH treatment and unchanged with alendronate administration. Patients treated with PTH had reduced body weight after 12 months, while those treated with alendronate showed no significant changes. In addition, alterations in ucOC were positively correlated with changes in adiponectin, but no association was found with insulin, glucose, leptin, or the insulin/glucose ratio. On the contrary, no significant difference in ucOC level was detected during treatment with risedronate [139].

Secondary osteoporosis is also an important health problem, the most common form being glucocorticoid-induced osteoporosis (GIO). Impaired bone formation is a central pathophysiological mechanism of GIO-related bone loss [102]. At the molecular level, excess glucocorticoids inhibit Wnt protein production, causing mesenchymal progenitor cells to differentiate into adipocytes rather than osteoblasts. Excessive glucocorticoids also interfere with the canonical BMP (bone morphogenetic protein) pathway [142]. Consequently, serum tOC levels were reduced in patients receiving glucocorticoids, even when administered at lower doses [143, 144]. In the study by Shikano et al. [145], serum ucOC levels showed a decrease during glucocorticoid treatment from the first week and remained decreased for four weeks. Menatetrenone treatment improved tOC levels in the third and fourth weeks.

## Osteocalcin and osteopetrosis

Osteopetrosis is a group of rare bone disorders within the family of sclerosing bone dysplasias, characterized by reduced bone resorption leading to high bone mass. An overly dense bone architecture, instead of providing strength, negates structural fragility that predisposes to fractures. Disruption of normal bone remodeling can lead to skeletal deformation and can interfere with mineral homeostasis [146]. Interestingly, mice lacking OC have severe osteopetrosis [147]. In general, three different types of osteopetrosis can be classified in humans: autosomal recessive (ARO), intermediate recessive (IRO) and autosomal dominant (ADO). They are characterized by different modes of inheritance and severity, from asymptomatic to fatal [11, 148].

Overall, individuals with ADO had increased level of tOC in cortical bone [149], reduced ucOC/tOC ratio (Table 1) and hypoinsulinemia [150]. In osteopetrotic children, serum OC levels were reported as normal or low. However, in a subset of six children, osteoblast activity (assessed by circulating ALP and OC) and osteoblast number did not increase despite hyperparathyroidism, suggesting PTH resistance or defective osteoblasts [151].

Osteopetrosis can be generally treated with calcium and vitamin D (first-line therapy), calcitriol, red blood cell transfusion, interferon γ-1b, and corticosteroids [146], however, there is no known link between these medications and OC level.

## Osteocalcin and inflammatory joint diseases

Inflammatory diseases affecting bones and joints are very common in the world, as 250 million and 14 million people worldwide suffer from osteoarthritis (OA) and rheumatoid arthritis (RA), respectively [152, 153]. Primary joint diseases are characterized by systemic osteoporosis and higher incidence of fractures [154]. These disorders are also associated with the presence of inflammatory process targeting the joints with adverse effects on their structure and function [155]. From this group, OA and RA are described in this review.

OA can be defined by joint symptoms, structural pathology, or both [156]. It is characterized by excessive cartilage degradation, abnormal bone growth, sclerosis, and synovial inflammation [157].

Campion et al. [158] determined reduced tOC levels in patients with non-destructive OA and a non-significant increase in those with destructive OA. On the contrary, Naito et al. [159] revealed elevated ucOC levels in subjects with bilateral knee OA versus healthy controls. The data are summarized in Table 1. An increased OC expression in articular cartilage and subchondral bone was detected in OA joints [158, 160, 161]. Karsdal et al. [162] found a 12% reduction in tOC levels in patients with symptomatic knee OA treated with salmon calcitonin compared to untreated individuals; however, this decrease was transient and only a 2% decrease was noted at the end of the study (Table 2).

RA is a systemic inflammatory autoimmune disease characterized by synovial inflammation and hyperplasia, autoantibodies production, and systemic features (cardiovascular, pulmonary, and psychological disorders) [163]. It is also responsible for joint destruction consistent with bone complications, which include periarticular bone loss, bone erosion, and systemic osteoporosis [155].

Non-significantly lower tOC levels were found in patients with RA [158]. Seriolo et al. [164] reported decreased tOC levels in individuals with active RA compared to those without active disease (Table 1). Although the physiological significance of alterations in tOC levels in OA and RA is still unknown, they may reflect distinct bone phenotypes between these diseases [1]. While OA is more characterized by an increase in subchondral bone mass, RA involves bone resorption and subchondral bone loss [164, 165].

According to Peretz et al. [166], tOC levels have not been shown to be a useful index of abnormal bone turnover in RA, except in some patients with vertebral fractures treated with low doses of corticosteroids. No significant differences in tOC levels were recorded in individuals with RA treated with low-dose corticosteroids or non-steroidal antiinflammatory drugs and untreated controls (Table 2). In the study by Tamai et al. [167], tOC levels in RA patients with erosion progression were significantly increased in the tocilizumab (anti-interleukin-6 receptor antibody)-treated group than in the infliximab (anti-tumor necrosis factor drug)-treated group.

## Conclusions and perspectives

Three forms of OC (cOC, ucOC, tOC) can be generally determined. In clinical conditions, ucOC/tOC ratio is often measured because it allows early identification of individuals at risk of low physical function and thus prevent future falls. Overall, cOC is required for optimal bone strength, ucOC is considered an endocrinologically active form, and tOC is regarded a marker of bone turnover. The vast majority of information on OC is limited to *in vitro* and animal model studies and therefore may not accurately reflect the situation in humans. Moreover, most clinical trials investigated groups pre-defined according to age, ethnicity or gender, which may also distort the data obtained. Limitations of existing knowledge also include technical problems with commonly used serum ucOC assays. Therefore, the medical-scientific community must continue efforts to elucidate the involvement of ucOCs in human health and disease and its clinical applications.

In addition to current knowledge of OC, this review also described its role in the management of DM, osteoporosis, osteopetrosis, OA and RA, focusing mainly on available clinical studies. Significantly reduced levels of tOC and ucOC could be associated with the risk of T2DM. The role of tOC and ucOC in T1DM remains uncertain. The tOC level does not seem to be a good indicator of high bone turnover status in PMO, OA and RA due to controversial findings. It is also worth noting that the correlation does not imply a direct function of OC in humans, so investigation of larger patient cohorts as well as carefully performed genetic studies are needed to gain more mechanistic insights. Additionally, associations between several pharmacological drugs used to treat all aforementioned bone-related disorders and OC levels were revealed. From this point of view, OC may serve as a medium through which some medications can affect glucose metabolism (e.g. glucocorticoids, teriparatide), body weight (e.g. PTH), adiponectin secretion (e.g. PTH), and synovial inflammation (e.g. tocilizumab). Last but not least, this review can contribute to a better use of OC in clinical applications as well as in further research and thus enrich current scientific literature.

## Data Availability

No original datasets were generated for this review. All data supporting the information given here can be found in the references cited within the paper.
